# Selective activation of miRNAs of the primate-specific chromosome 19 miRNA cluster (C19MC) in cancer and stem cells and possible contribution to regulation of apoptosis

**DOI:** 10.1186/s12929-017-0326-z

**Published:** 2017-03-07

**Authors:** Phan Nguyen Nhi Nguyen, Chiu-Jung Huang, Shigeki Sugii, Soon Keng Cheong, Kong Bung Choo

**Affiliations:** 1 Centre for Stem Cell Research, Universiti Tunku Abdul Rahman, 43000 Kajang, Selangor Malaysia; 2 Postgraduate Program, Universiti Tunku Abdul Rahman, 43000 Kajang, Selangor Malaysia; 3 Department of Animal Science & Graduate Institute of Biotechnology, Chinese Culture University, Taipei, Taiwan; 4 Singapore BioImaging Consortium, A*Star, Singapore, Singapore; 5 Duke-NUS Graduate Medical School, Singapore, Singapore; 6 Department of Preclinical Sciences, Faculty of Medicine and Health Sciences, Universiti Tunku Abdul Rahman, 43000 Kajang, Selangor Malaysia; 7 Department of Preclinical Sciences, Faculty of Medicine and Health Sciences, Center for Stem Cell Research, Universiti Tunku Abdul Rahman, Sungai Long campus, Bandar Sungai Long, Cheras, 43000 Kajang, Selangor Darul Ehsan Malaysia

**Keywords:** C19MC, Cancer, Apoptosis, miRNA expression, Stem cells

## Abstract

**Background:**

The human chromosome 19 miRNA cluster (C19MC) of 43 genes is a primate-specific miRNA cluster that may have biological significance in the genetic complexity of the primate. Despite previous reports on individual C19MC miRNA expression in cancer and stem cells, systematic studies on C19MC miRNA expression and biological functions are lacking.

**Results:**

Cluster-wide C19MC miRNA expression profiling by microarray analysis showed wholesome C19MC activation in embryonic stem cells (ESCs) and induced pluripotent stem cells (iPSCs). However, in multipotent adipose-derived mesenchymal stem cells (MSCs) and a unipotent human white pre-adipocyte cell line, only selected C19MC miRNAs were expressed. MiRNA copy number analysis also showed selective C19MC expression in cancer cells with expression patterns highly similar to those in MSCs, suggesting similar miRNA regulatory mechanisms in these cells. Selective miRNA expression also suggests complex transcriptional mechanism(s) regulating C19MC expression under specific cellular and pathological conditions. Bioinformatics analysis showed that sixteen of the C19MC miRNAs share the same “AAGUGC” seed sequence with members of the miR-302/-372 family, which are known cellular reprogramming factors. In particular, C19MC-AAGUGC-miRNAs with the nucleotides 2-7 canonical seed position as in miR-302/-372 miRNAs, may play similar roles as miR-302/-372 in induced pluripotency. A biased 3p-arm selection of the C19MC-AAGUGC-miRNAs was observed indicating that targets of the 3p species of these miRNAs may be biologically significant in regulating stemness. Furthermore, bioinformatics analysis of the putative targets of the C19MC-AAGUGC-miRNAs predicted significant involvement of signaling pathways in reprogramming, many of which contribute to promoting apoptosis by indirect activation of the pro-apoptotic proteins BAK/BAX via suppression of genes of the cell survival pathways, or by enhancing caspase-8 activation through targeting inhibitors of TRAIL-inducing apoptosis.

**Conclusions:**

This work demonstrated selective C19MC expression in MSCs and cancer cells, and, through miRNA profiling and bioinformatics analysis, predicted C19MC modulation of apoptosis in induced pluripotency and tumorigenesis.

**Electronic supplementary material:**

The online version of this article (doi:10.1186/s12929-017-0326-z) contains supplementary material, which is available to authorized users.

## Background

MicroRNAs (miRNAs) are short noncoding single-stranded RNAs that act post-transcriptionally as negative regulators of gene expression. In the miRNA biogenesis process, either or both 5p and 3p miRNA species may be generated from the pre-miRNA precursor arms [1, 2]. Selective maturation or co-existence of the 5p and 3p species is biologically important since they target different gene sets. MiRNAs also form family groups defined by short homologous sequences, called the seed sequence, which is located at the 5’- end of the mature miRNAs. MiRNAs of the same family often form functionally-related groups that cross-regulate targets to ensure conservation of biological functions [3, 4].

miRNAs are involved in regulating developmental patterning, maintenance of stem cell self-renewal and cancer progression [5, 6]. Specific miRNAs are up-regulated in pluripotent stem cell population but not in mature differentiated cell types in early embryonic development [7]. Specific miRNAs have also been shown to be able to reprogram somatic cells to induced pluripotent stem cells (iPSCs) [8, 9]. MiR-302-driven cellular reprogramming coordinates stem cell division by regulating targets in the cell cycle, particularly at the G1/S restriction point [5]. Besides the roles in stem cell biology, miRNAs also act as tumor suppressors or oncogenes in the tumorigenesis process [10–14]. Thus, aberrant expression of miRNA affects crucial processes in the development and progression of tumors, including induction of anti-apoptosis, development of drug resistance and cancer invasion and metastasis [15–22].

For regulatory advantages, miRNAs, particular those from the same family, are often clustered in specific chromosomal locations [23]. One such human miRNA cluster is mapped on chromosome 19, and is called the chromosome 19 miRNA cluster, or C19MC [24]. C19MC, one of the largest miRNA gene clusters in the human genome, contains 46 highly homologous miRNA genes, including 7 duplicated pairs of the same genes, within a ~100-kb genomic region. Importantly, C19MC is a primate-specific miRNA cluster that appeared late in the evolution of the primate lineage; bioinformatics analysis has predicted that C19MC miRNAs play critical roles in reproduction, development and differentiation in the primate compared to the lower vertebrates [25]. Reproduction-related role of C19MC is further reflected in its restrictive expression in reproductive tissues, but not in other adult organs and tissues; C19MC expression has also been shown in pluripotent embryonic stem cells (ESCs) [7, 11, 25]. In the human placenta, C19MC is expressed *en bloc* from the paternal allele governed by a major promoter located 17.6 kb upstream of the first miRNA gene in the cluster [26]. Transcription of the C19MC cluster was further suggested to be mediated by demethylation of the upstream CpG-rich master promoter region to first generate a primary transcript encompassing the entire C19MC gene cluster, followed by splicing to generate the individual precursor miRNA species, and subsequently processed by the DGCR8-Drosha microprocessor complex to generate individual mature miRNAs [24]. The implication of a master promoter mode of transcription is the all-or-none presence of C19MC miRNAs in the expressed cells. At present, the biological functions and expression patterns of C19MC members in other stem cell types and in cancer cells have not been systematically examined in a cluster-wide manner.

In a previous study, we reported genome-wide miRNA profiling analysis of ESCs, iPSCs and mesenchymal stem cells (MSCs), proposing cross- and co-regulation by 5p and 3p paired miRNA species during reprogramming [4]. Using the same miRNA microarray profiling dataset, we focused in this work on the expression profiles of C19MC miRNAs in various stem cell types and in cancer cells. Possible biological functions of a subset of miR-302-like C19MC miRNAs, were further investigated by bioinformatics analysis, which predicted targeting at the apoptosis pathway in the tumorigenesis of cancer cells and induced pluripotency in stem cells.

## Methods

### Cell lines

We have previously described iPSC lines derived from two MSCs, namely adipose stem cell (ASC; Invitrogen, Carlsbad, CA, USA) and human adipose-derived MSC (MSC-AT; PromoCell, Heidelberg, Germany), and from a human white pre-adipocyte (HWP) cell line [4, 27]. In this work, human adipose-derived MSC, designated ASC Lonza, was purchased from Lonza, Lonza, Verviers, Belgium. MH#1 was an iPSC cell lined established from ASC Lonza in our lab (S. Sugii, unpublished data). WJ0706 is a human MSC cell line derived from Wharton’s Jelly (WJ) obtained from Cytopeutics Sdn. Bhd, Selangor, Malaysia (http://www.cytopeutics.com). The MSC cell lines were isolated and characterized at Cytopeutics according to standard procedures and with ethical clearance [28]. Human placenta choriocarcinoma cell line JEG-3 (ATCC HTB-36), human normal placental cell line HS 799. PI (ATCC CRL-7530) and human normal colon cell line CRL-1790 (ATCC CRL-1790) were purchased from ATCC (Manassas, VA, USA). Cancer cell lines were kindly provided by Professor Y.M. Lim, Cancer Research Center, Universiti Tunku Abdul Rahman.

### miRNA microarray profiling

Total RNA was isolated from the cell lines by using the MiRNeasy Mini Kit (Qiagen, USA) according to the manufacturer’s manual. Microarray analysis was performed using the SmartChip Human MicroRNA Panel version 3.0 (WaferGen Biosystems, Fremont, CA, USA) containing 1036 unique real-time PCR reactions in quadruplicates as previously described [4]. To identify differentially expressed miRNAs, the iPSC data were compared with data of the parental MSC or HWP cells from which they were derived. For calculation of expression levels, the All-Mean Normalization method was employed, where mean C_t_ values of all expressed genes were used. To compute the expression levels of expressed miRNAs, the C_t_ values of each sample were compared to its average C_t_ (All-Mean) to obtain the ΔC_t_ values. ΔΔC_t_ was then calculated by the two ΔC_t_ values between the iPSC and its parental cells. Log_2_(fold change), or log_2_(FC), was computed as log_2_[FC (2^-ΔΔCt^)]. The selection criteria for differentially expressed miRNA was log_2_(FC) > 1.5 or < -1.5, with *p* < 0.05 as determined by the Student’s *t* test.

### Reverse transcription and miRNA real-time PCR assays

RNA was reverse-transcribed using the TaqMan MicroRNA Reverse Transcription Kit and miRNA-specific stem-loop primers (Applied Biosystems, USA). The reverse transcription reaction was as follows: 0.075 μl of 100 mM dNTP, 0.5 μl RT enzyme (50 U/μl), 0.75 μl 10X RT buffer, 0.094 μl RNAse inhibitor (20 U/μl), 50 ng RNA. Reaction conditions were 16 °C for 30 min, 42 °C for 30 min, 85 °C for 5 min, and held at 4 °C. Real-time PCR assays of the transcribed cDNA were performed using the TaqMan MicroRNA assays (Applied Biosystems, USA). The reaction mixture was as follows: 10 μl TaqMan 2X universal PCR master mix, 1 μl 20X TaqMan MicroRNA Assay, 1.33 μl cDNA and 7.67 μl nuclease-free water. Reaction conditions were: 95 °C for 10 min, followed by 40 cycles at 95 °C for 15 s, and 60 °C for 1 min.

### Determination of absolute copy number of mature miRNAs

Synthetic mature miRNAs (Integrated DNA Technologies IDT, Coralville, IA, USA) were serially diluted to final concentrations of 200 nM, 20 nM, 2 nM, 0.2 nM, 0.02 nM, 2 pM, 0.2 pM and 0.02 pM. Serially-diluted synthetic RNAs were reverse-transcribed and subjected to real-time PCR analysis concurrently with the sample RNAs. Standard curves were included on each plate of the miRNA TaqMan assays to convert the cycle threshold (C_t_) values of each sample into the corresponding number of miRNA copies in each cell, assuming that each cell contains 15 pg total RNA, as previously described [29]. C_t_ values ≥ 35 indicated that their expression levels were too low for accurate analysis, and were considered no detectable expression. The cut-off threshold of miRNA expression was, therefore, standardized at C_t_ < 35.

### Construction of phylogeny tree

The stem-loop sequences for C19MC were downloaded from miRBase ver. 21. A phylogenetic tree was generated by multiple sequence alignment using the Clustal method of the Megalign project provided by DNAstar® (Salt Lake City, Utah, USA).

### Prediction of miRNA target genes

miRNA:mRNA interactions were predicted using the major miRNA databases TargetScan and microRNA.org. To identify genes and pathways targeted specifically by selected C19MC-AAGUGC-miRNAs, overlapping target gene sets of the selected miRNAs were used for the Kyoto Encyclopedia of Genes and Genomes (KEGG) pathway and Gene Ontology (GO) annotation analysis based on the web-based DAVID (Database for Annotation, Visualization and Integrated) algorithm. The criteria of analysis was EASE score ≤ 0.05, in which EASE score is a modified Fisher Exact *P* value in the DAVID system used for gene-enrichment analysis. An EASE score *P* value = 0 represents perfect enrichment; *P* value ≤ 0.05 was considered as significant gene-enrichment in a specific annotation category.

## Results

### Selective activation of C19MC miRNAs in mesenchymal stem cells

**Fig. 1:**
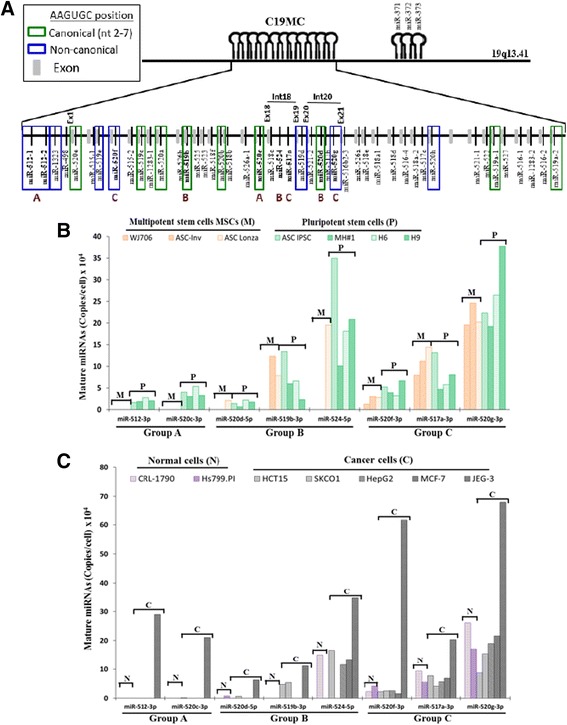
Expression of selected C19MC miRNAs in different cell lines. **a** A scheme displaying relative genomic locations of the C19MC and the miR-372 family miRNAs on human chromosome 19q13.41. MiRNAs in green and blue boxes harbor the AAGUGC seed sequence in the canonical (nts 2-7) or non-canonical positions, respectively (see Fig. 2a). The proposed exon sequences (Ex) of the clusters (Bortolin-Cavaille et al. [24]) are shown in short gray bars between the miRNAs; introns (Int) 18 and 20 shown carry two of multiple miRNA genes analyzed. The eight C19MC miRNAs selected for expression analysis in (**b**) and (**c**) below are shown in *bold*, with the expression A-C grouping designations established in (**b**) and (**c**) shown at the *bottom* of the miRNAs. **b** Expression of selected C19MC miRNAs, determined based on copy number per cell, in mesenchymal stem cells (MSCs). The MSCs included are: MSC-AT, WJ0706, ASC-INV and ASC. ASC IPSC and MH#1 are iPSCs derived from ASC-INV and ASC Lonza, respectively. H6 and H9 are human ESC cell lines. **c** C19MC miRNA expression in cancer cells. The cell lines used are: CRL-1790: normal colon cells, Hs799. PI: normal placenta cells, HCT15 and SK-CO-1: colorectal cancer cells; HepG2: hepatocellular carcinoma cells, MCF7: breast cancer cells; JEG3: choriocarcinoma cells. C_t_ values ≥ 35 was used as the cut-off threshold in the analysis

We have previously reported genome-wide miRNA expression profiling of two ESCs, two multipotent adipose-derived MSCs, a unipotent HWP cell line, and the three iPSC lines derived from the MSCs and HWP [4]. In this study, the expression data of C19MC miRNAs in the MSCs and HWP, and the MSC- and HWP-derived iPSCs were extracted for further analysis. Our results showed that all the forty-five C19MC miRNAs included in the microarray in either the 5p or 3p or in both 5p/3p configurations were expressed, albeit to different extents, in all the three pluripotent iPSC cell lines tested (Table 1), and in the hESC controls (data not shown). The miR-372 family that lies adjacent to the C19MC cluster (Fig. 1a) was also included in the analysis since they have been reported to be expressed in pluripotent stem cells [9]. Of the forty-five C19MC miRNAs, thirty-nine were significantly (*p* < 0.05) expressed, as previously reported [7]. Expression of the C19MC miRNAs in the iPSCs was generally two-fold or greater than that in the parental cell lines; the highest level of expression was 8.375 log_2_(fold change) in miR-520b (Table 1). Notably, both the 5p and 3p miRNA species were expressed in most cases; otherwise, the 3p species was the favored precursor arm selected for the mature miRNAs, as opposed to frequent 5p arm expression in most other miRNA genes [2].

**Table 1:** Expression of C19MC and the miR-372 family miRNAs in stem cells

miRNA^a^	Gene copy	Expression^b^			Log_2_(fold change)
		HWP	MSC	iPSC	
miR-512-5p	2	-	+/-	+	5.265 ± 0.58**
miR-512-3p		+	+	+	3.600 ± 1.85*
miR-1323 (5p)	1	-	-	+	7.319 ± 0.50**
miR-498 (5p)	1	+	+	+	3.857 ± 0.99**
miR-520e (3p)	1	-	-	+	4.001 ± 0.20**
miR-515-5p	2	-	-	+	7.053 ± 0.63**
miR-515-3p		-	-	+	4.083 ± 0.45**
miR-519e-5p	1	-	-	+	3.046 ± 0.55**
miR-519e-3p		-	-	+	3.320 ± 0.44**
miR-520f-3p	1	-	-	+	6.275 ± 0.75**
miR-519c-3p	1	-	-	+	5.685 ± 0.66**
miR-1283 (5p)	2	-	-	+	3.055 ± 0.29**
miR-520a-5p	1	-	-	+	4.863 ± 0.71**
miR-520a-3p		+	+	+	0.869 ± 1.48
miR-526b-5p	1	-	+	+	4.233 ± 0.13*
miR-526b-3p		-	+/-	+	6.906 ± 0.81**
miR-519b-3p	1	-	+	+	2.620 ± 1.36*
miR-518f-5p	1	-	+/-	+	3.901 ± 0.50**
miR-518f-3p		-	+/-	+	6.971 ± 0.59**
miR-520b (3p)	1	-	-	+	8.375 ± 0.54**
miR-518b (3p)	1	+	+	+	3.706 ± 0.98*
miR-526a (5p)	2	-	+	+	2.741 ± 1.95
miR-520c-3p	1	-	-	+	8.285 ± 0.70**
miR-518c-5p	1	n.d.	-	+	4.135 ± 0.34
miR-518c-3p		-	-	+	6.282 ± 0.76**
miR-524-5p	1	-	-	+	3.810 ± 0.60**
miR-524-3p		-	-	+	4.202 ± 0.63**
miR-517a-5p	1	-	+/-	+	2.328 ± 0.65*
miR-517a-3p		-	-	+	8.262 ± 0.62**
miR-519d-3p	1	-	+/-	+	4.819 ± 2.56*
miR-521 (3p)	2	+	+	+	1.500 ± 0.30*
miR-520d-5p	1	-	-	+	3.933 ± 0.34**
miR-520d-3p		n.d.	+	+	0.233 ± 3.93
miR-517b-3p	1	-	+/-	+	6.474 ± 0.73**
miR-520g-3p	1	+	+	+	6.014 ± 1.27**
miR-516b-5p	2	-	-	+	3.243 ± 0.52
miR-518e-5p	1	-	+	+	3.344 ± 1.88
miR-518e-3p		+	+	+	1.386 ± 0.49*
miR-518a-3p	2	+	+	+	2.013 ± 1.04*
miR-518d-3p	1	-	-	+	3.613 ± 0.47**
miR-520h (3p)	1	-	+	+	5.618 ± 0.82**
miR-522-3p	1	-	-	+	4.060 ± 0.47**
miR-519a-3p	2	-	-	+	6.586 ± 0.56**
miR-516a-5p	2	-	-	+	3.583 ± 0.32**
miR-516a-3p		-	+/-	+	0.758 ± 0.52*
miR-371a-5p	1	-	-	+	6.256 ± 1.14**
miR-372a-3p		+	+/-	+	1.094 ± 5.34
miR-372-3p	1	+	+	+	7.201 ± 0.11**
miR-373-5p	1	-	-	+	2.500 ± 1.27*
miR-373-3p		-	-	+	7.240 ± 1.39**

^a^miRNA-5p and -3p designations are based on miRBase ver. 21; -5p and -3p designations in brackets are not annotated in miRBase, but are the presumptive precursor arms derived from sequence alignment. miRNAs are arranged in order of relative physical locations on chromosomae 19.13q.41; the neighbouring miR-371-3 cluster is also shown. ^b^The two MSC cell lines were used in comparison with the three iPSC lines derived. “+” and “-” indicate detectable and undetectable expression of the miRNA, respectively, in both cell lines; “+/-” indicates that one of the two MSC was positive and the other one was negative. *n.d.* not done

*p < 0.05, **p < 0.01

**Fig. 2:**
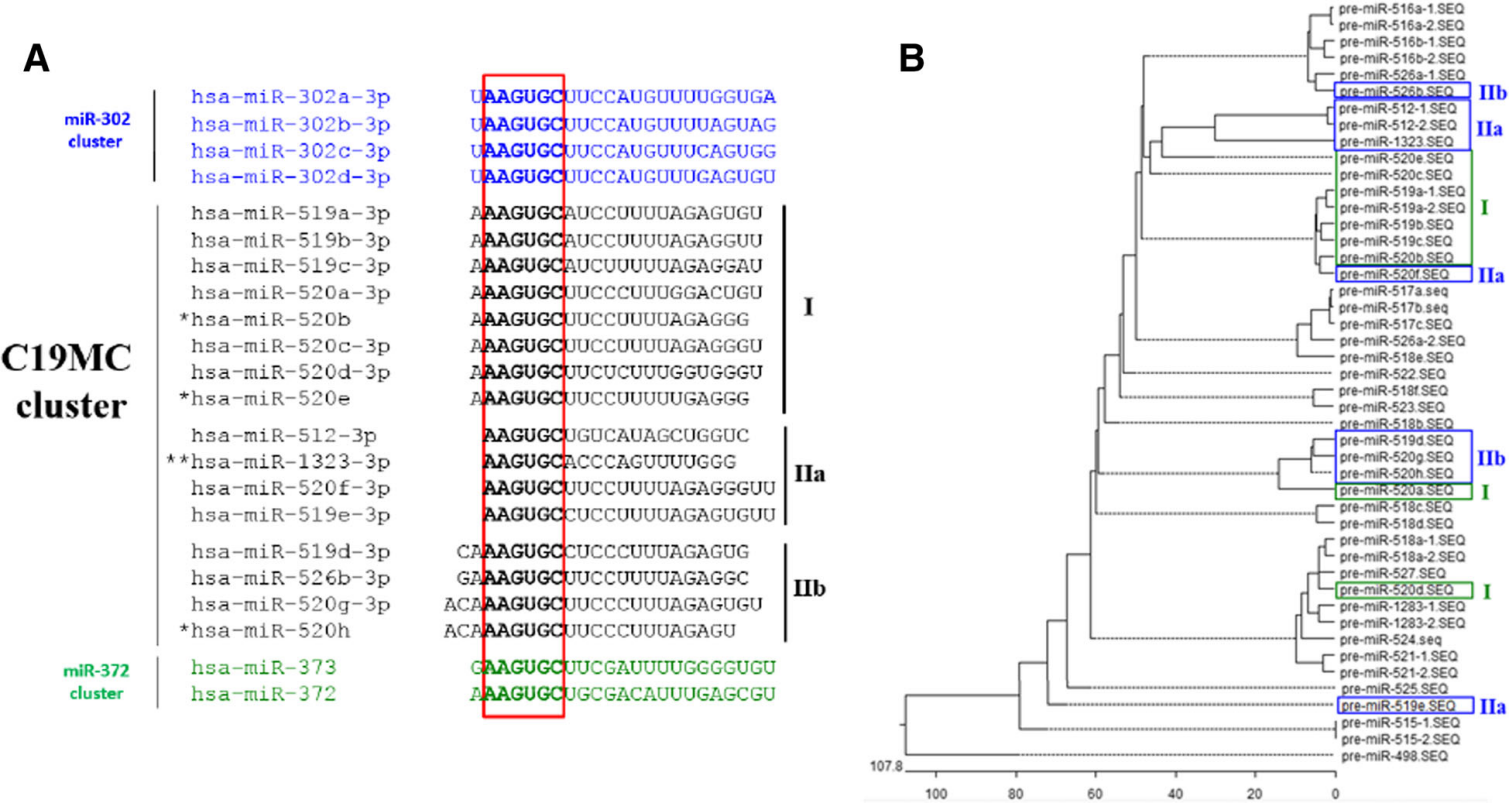
C19MC miRNAs harboring the AAGUGC hexameric sequence. **a** The sixteen C19MC miRNAs that share the AAGUGC hexameric seed sequence (in *bold letters* and boxed in *red*) with the miR-302 (in *blue letters*) and miR-372 (in *green letters*) families are shown. MiRNAs that have the AAGUGC seed sequence in the canonical nucleotides 2-7 position are called group I; other miRNAs in non-canonical position are called group II, with IIa and IIb subgroups as depicted. *miRNAs are in the 3p configuration as in the miRBase ver 21. **The existence of miR-1323-3p is based on computational prediction. **b** Phylogenic tree of all C19MC miRNAs reconstructed with the precursors of the miRNAs. Groups I, IIa and IIb are AAGUGC-harboring miRNAs as defined in (**b**) above (see text for further description)

On the other hand, only selected C19MC miRNAs were found to be expressed in MSC and HWP (Tables 1 and 2). Many of the expressed miRNAs share the “AAGUGC” seed sequence of the known reprogramming miR-302 miRNA family; these miRNAs are called the C19MC-AAGUGC-miRNAs in this work (see Fig. 2a and depiction below). Twenty-two (48.9%) of the forty-five C19MC miRNAs were activated in one or both MSC cell lines. Only eight miRNAs were expressed in HWP, which were, interestingly, also all expressed in the two MSC and all pluripotent cells (Table 2). This may suggest that these eight miRNAs constitute the minimal miRNA set require for minimal potency in the unipotent HWP. Thus, the cluster-wide microarray results indicated selective activation of twenty-two C19MC miRNAs in multipotent mesenchymal stem cells.

**Table 2:** Expression of C19MC miRNAs in different stem cell types

Stem cell type	Potency	miRNA^a^		No.
		AAGUGC seed sequence^b^	Others	
iPSC/hESC	Pluripotent	16 miRNAs	29 miRNAs	45
MSC	Multipotent			
One cell line		miR-519d-3p, miR-526b-3p	miR-512-5p, miR-516a-3p, miR-517a-5p, miR-517b-3p, miR-518f-5p, miR-518f-3p	8
Both cell lines		miR**-512-3p**, **miR-519b-3p**, miR-520a-3p, miR-520d-3p, **miR-520g-3p**, miR520h	miR-498, miR-518a-3p, miR-518b, miR-518e-5p, miR-518e-3p, miR-521, miR-526a, miR-526b-5p	14
HWP	Unipotent	miR-520a-3p, **miR-520g-3p**, **miR-512-3p**	miR-498, miR-518a-3p, miR-518b, miR-518e-3p, miR-521	8
All cell lines	Pluri-/multi-/unipotent	miR-520a-3p, **miR-520g-3p**	miR-498, **miR-512-3p**, miR-518a-3p, miR-518b, miR-518e-3p, miR-521	8

^a^miRNAs in bold letters were used for further quantification as depicted in Fig. 1. ^b^AAGUGC seed sequence-containing miRNAs are taken from Fig. 2

It has been reported that C19MC miRNAs are not expressed in adult tissues except in tissues of the reproductive system [11]. To obtain further supporting evidences on selective activation, expression of eight miRNAs spanning the C19MC cluster (Fig. 1a), but with different genomic structures, was selected for further experimentally verification; amongst the selected miRNAs, miR-512-3p is transcribed by the two miR-512-1 and-512-2 genes located at the 5’-end of the C19MC miRNA gene cluster; miR-520c-3p, -519b-3p and -520f-3p are single miRNA genes located between previously proposed exons; miR-524-5p and -517a-3p are two of three miRNA genes mapped on intron 18 and miR520d-5p and -520g-3p are two of four miRNAs mapped on intron 20 (Fig. 1a) [24]. Verification was done in three other different MSC cell lines, namely the MSC cell line WJ0706 derived from the Wharton’s Jelly [28], and two other adipose-derived MSC cell lines, ASC-Inv and ASC Lonza (Fig. 1b). In the experiments, two other adipose MSC-derived iPSCs, ASC-iPSC and MH#1, and two hESCs, H6 and H9, were included. The miRNA expression levels were determined as the absolute miRNA transcript copy number per cell, which ranged from 0 copy, at a real-time RT-PCR C_t_ value ≥ 35 (see Methods), to 377,200 copies per cell at a C_t_ value of 25.7 in miR-520g-3p in ESC H9 cells **(**Fig. 1b).

Consistent with the miRNA microarray results, the selected miRNAs were all expressed to different levels in all four iPSCs and ESCs (Fig. 1b). In contrast, the tested miRNAs were either not expressed, or expressed to different but lower levels in the MSCs tested. MSC expression of the eight C19MC miRNAs could be grouped in three expression patterns: group A, which included miR-512-3p and -520c-3p, showed very low or undetectable expression in the MSCs; expression of the group B miR-520d-5p, 519b-3p and -524-5p was detected in at least one or both MSC cell lines, whereas miR-520f-3p, -517a-3p and -520g-3p in group C were all expressed all three MSC cell lines (Fig. 1b and Table 2). The collective results obtained from the microarray and real-time RT-PCR experiments, therefore, confirmed selective C19MC expression in multipotent MSCs, and *en bloc* expression in pluripotent iPSCs. Furthermore, there seemed to be no correlation between the expression pattern and the physical location of the miRNA genes tested (Fig. 1a). Notably, the miR-524-5p and -517a-3p and the miR520d-5p and -520g-3p couples are flanked by two proposed exons but belong to different expression groups B and C (Fig. 1a). The data suggest regulation by different promoters or transcriptional regulatory mechanism(s) other than simple splicing of the two flanking exon and co-processing of the spliced intron sequence as previously proposed for C19MC expression in a choriocarcinoma JEG-3 cell line [24]. The observation further suggests a critical biological role of the expressed C19MC miRNA in conferring different degrees of stemness to the stem cells, particularly in MSCs.

### Selective activation of C19MC miRNAs in cancer cells

Previous reports have indicated frequent activation of C19MC miRNAs in different cancer types, including colorectal cancer, breast cancer and primitive neuroectodermal brain tumor [13, 30, 31] (see below). To investigate C19MC expression in cancer cells, the expression of the same set of eight C19MC miRNAs was also quantified as gene copy number per cell in two colorectal cancer (HCT15 and SKCO1), one breast cancer (MCF-7) and one hepatocellular carcinoma (HepG2) cell lines; the choriocarcinoma (JEG-3) cell line, which was derived from the reproductive system, was included a positive control since JEG-3 cells have been shown to express all C19MC miRNAs in high levels [26] (Fig. 1c). Two cell lines CRL-1790 and HS799. PI, derived from normal colon and placenta tissues, respectively, were also included in the analysis. Despite *en bloc* and high-level C19MC expression in JEG-3 cells, only four of the eight miRNAs, namely miR-520d-5p of Group B as defined above for stem cell expression, and all three Group C miRNAs, miR-520f-3p, -517a-3p and -520g-3p, were shown to be expressed in the normal placenta cell line Hs799. PI. Furthermore, expression of the Group B miR-524-5p, and all three Group C miRNAs was detected in CRL-1790, which was derived from normal fetal colon epithelium (Fig. 1c). The observed expression of selective C19MC in fetal colon epithelium and in the placenta is consistent with previous conclusions that C19MC is specifically expressed in reproduction and developmental process-related tissues and is silenced in normal tissues [7, 11, 25]. Interestingly, in the five cancer cell lines examined, the selective expression patterns of the eight miRNAs was similar those shown in MSCs above (Fig. 1b). Group A miRNAs also showed very low or undetectable expression in normal and cancer cells, except in JEG-3, whereas the Group B miRNAs were detected in one or more cancer cell lines; all three Group C miRNAs were expressed all four cancer cell lines (Fig. 1c). Taken together, quantitative expression analysis showed highly similar C19MC miRNA expression profiles found in MSCs and cancer cells, suggesting that the C19MC miRNAs may share some similar molecular and biological features in transcriptional regulation and in the etiological pathways in acquiring multipotency and cancer phenotype.

### Identification of C19MC miRNAs harboring the “AAGUGC” seed sequence

miRNA-mRNA interactions involve the seed region at the 5’ end of the miRNA; hence, seed sequences are important predictors for the identification of miRNA-targeted transcripts [1]. MiRNAs that share a common seed sequence also might share target specificity and possibly biological functions. On sequence alignment, sixteen C19MC miRNAs were found to share the same seed sequence, 5’-AAGUGC-3’, with the reported reprogramming-able miR-302 and miR-372 miRNA families [8, 9] (Fig. 2a). These miRNAs are designated as “C19MC-AAGUGC-miRNAs”. Furthermore, it is noted that the AAGUGC seed position at 5’ end is variable among the C19MC-AAGUGC-miRNAs: subgroup I miRNAs, which includes eight miR-519 and -520 subfamilies, have the seed sequence located at the canonical and optimal 5’-nucleotide positions (nts) 2-7, as in the miR-302/-372 families; the seed sequence of the four subgroup IIa miRNAs is at location nts 1-6, and that of the remaining subgroup IIb miRNAs is at nts 3-8 and 4-9 (Fig. 2a). Hence, despite the presence of the AAGUGC seed sequence, it is more likely that the nts 2-7 canonical subgroup of the C19MC-AAGUGC-miRNAs may target genes that share similar functions as the miR-302/-372 miRNAs.

While the 5p arm of a pre-miRNA precursor is normally selected for maturation [2], it is noted that the C19MC-AAGUGC-miRNAs are predominantly derived from the 3p arm of the precursor miRNAs, hinting at an evolutionary bias in 3p selection with possible biological implications. Further supporting evidence of conservation of the C19MC-AAGUGC-miRNAs was derived from the construction of a phylogenetic tree of all precursor sequences of the C19MC miRNAs (Fig. 2b). Most C19MC-AAGUGC-miRNAs are grouped into the same cluster in the top half of the phylogenetic tree. Four of the remaining C19MC-AAGUGC-miRNAs form another cluster in the middle of the tree and the remaining two miRNAs are scattered in different branches in the lower half of the tree (Fig. 2b).

### Bioinformatics predictions of possible biological functions of group I C19MC-AAGUGC-miRNAs

**Fig. 3:**
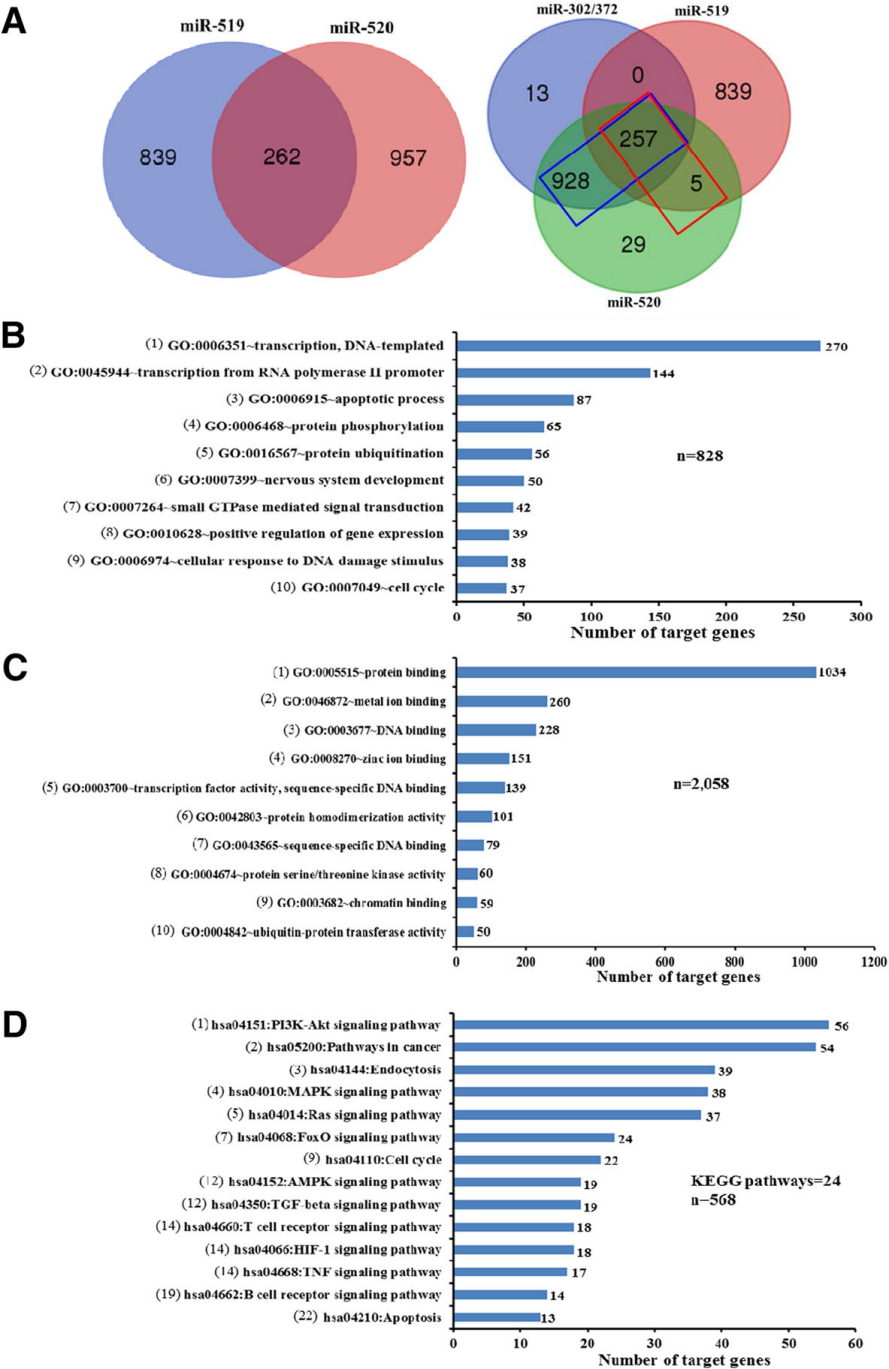
Bioinformatics analysis of predicted target genes of group I of the C19MC-AAGUGC-miRNAs. **a** Venn diagrams of predicted target genes of the miR-302/372 families and group I of the C19MC-AAGUGC-miRNAs. (*left panel*) The miR-519 and -520 subfamilies share only a small number of target genes. (*right panel*) The miR-520 miRNAs share a significant number of target genes with the miR-302/372 families. **b & c** The top 10 highest scores and the most significantly enriched GO terms associated with biological process and molecular function, respectively. **d** Fourteen of the 24 most enriched KEGG pathways displaying the ten signaling pathways identified; see text for explanation and Additional file 2: Table S2 for full list. The numerical in brackets shows the ranking of each pathway

It is noted that the C19MC-AAGUGC-miRNAs with the canonical nts 2-7 seed position, defined here as Group I (Fig. 2), contributed more significantly in gene targeting. Thus, in this study, we focused on analysis of potential biological functions of C19MC-AAGUGC-miRNAs in group I. Bioinformatics searches showed a total of 2058 putative target genes targeted by group I C19MC-AAGUGC-miRNAs (Fig. 3a and Additional file 1: Table S1). However, construction of a Venn diagram showed that only 262 putative target genes are common between the miR-519 and miR-520 subfamilies in group I, indicating that the miR-519 and -520 subfamilies target different sets of genes. The overlapping gene sets among miR-302/372 and the miR-519 and miR-520 subfamilies in group I were further compared (Fig. 3a). The results showed that 1185 putative shared genes were obtained between the miR-520 and -302/372 families (Fig. 3a, blue box and Additional file 1: Table S1), suggesting that the miR-520 subfamily might share similar biological functions with the miR-302/372 family. The group I miR-519 subfamily also shares 262 putative target genes with the miR-302/-372 families, far fewer than the miR-520 subfamily (Fig. 3a, red box). Consistent with the bioinformatics prediction, a literature review showed that a number of validated targets have indeed been reported to be shared between the miR-302/372 and the group I C19MC-AAGCGU-miRNA families (Table 3).

**Table 3:** Common validated target genes shared between the C19MC-AAGUGC-miRNAs and the miR-302/-372 families

AAGUGC-miRNA		Seed position^a^	Target transcript	References
miR-302/-372	C19MC			
miR-302c	miR-520e	I	NIK	[10, 15]
miR-373	miR-520c	I	MT1-MMP, mTOR, SIRT1	[14, 21]
miR-372, -373	miR-520c, -520e	I	RelA	[12]
miR-302b, -372, -373	miR-520c, -520e	I	TGFβR2	[9, 12]
	miR-520b, -520e	I	CD46	[16]
miR-302c	miR-520c	I	MICA, MICB, ULBP2	[17]
	miR-519a	I	RBL2	[13]
	miR-512	IIa		
	miR-519d, -520g	IIb	SMAD7	[19, 20]
	miR-520g, -520h	IIb	DAPK2	[18, 22]
miR-302d, -372	miR-520b, -519b-3p, -520a-3p	I	CDKN1A	[5, 6]
	miR-519e	IIa		
	miR-519d, -520h	IIb		

^a^Group I seed position is the canonical nts 2-7; IIa is nts 1-6 and IIb is other non-canonical position, as defined in Fig. 2a

The 2058 putative target genes were further subjected to GO analysis and KEGG pathway annotation (Fig. 3b-d). Of the 828 predicted targets in the top 10 GO terms in biological functions, 616 (74.4%) putative genes are associated with transcriptional and translational regulation of gene expression (Fig. 3b, GO terms 1, 2, 4, 5, 7 & 8). The remaining predicted targets regulate apoptosis, nervous system development, cellular response to DNA damage stimulus and cell cycle. The majority of the 2058 predicted genes in GO terms in molecular functions is likewise associated with transcriptional and translational regulation in some way (Fig. 3c), and in epigenetic regulation (Fig. 3c, GO term 9). Four hundred eleven genes (20.0%) are related to metal or zinc ion binding (Fig. 3c, GO terms 2 & 4). which may also be components of signaling pathways. Taken together, the GO analysis data suggested that the group I C19MC-AAGUGC-miRNAs are mainly associated with the regulation of gene expression, cell proliferation and apoptosis via various signaling pathways.

**Fig. 4:**
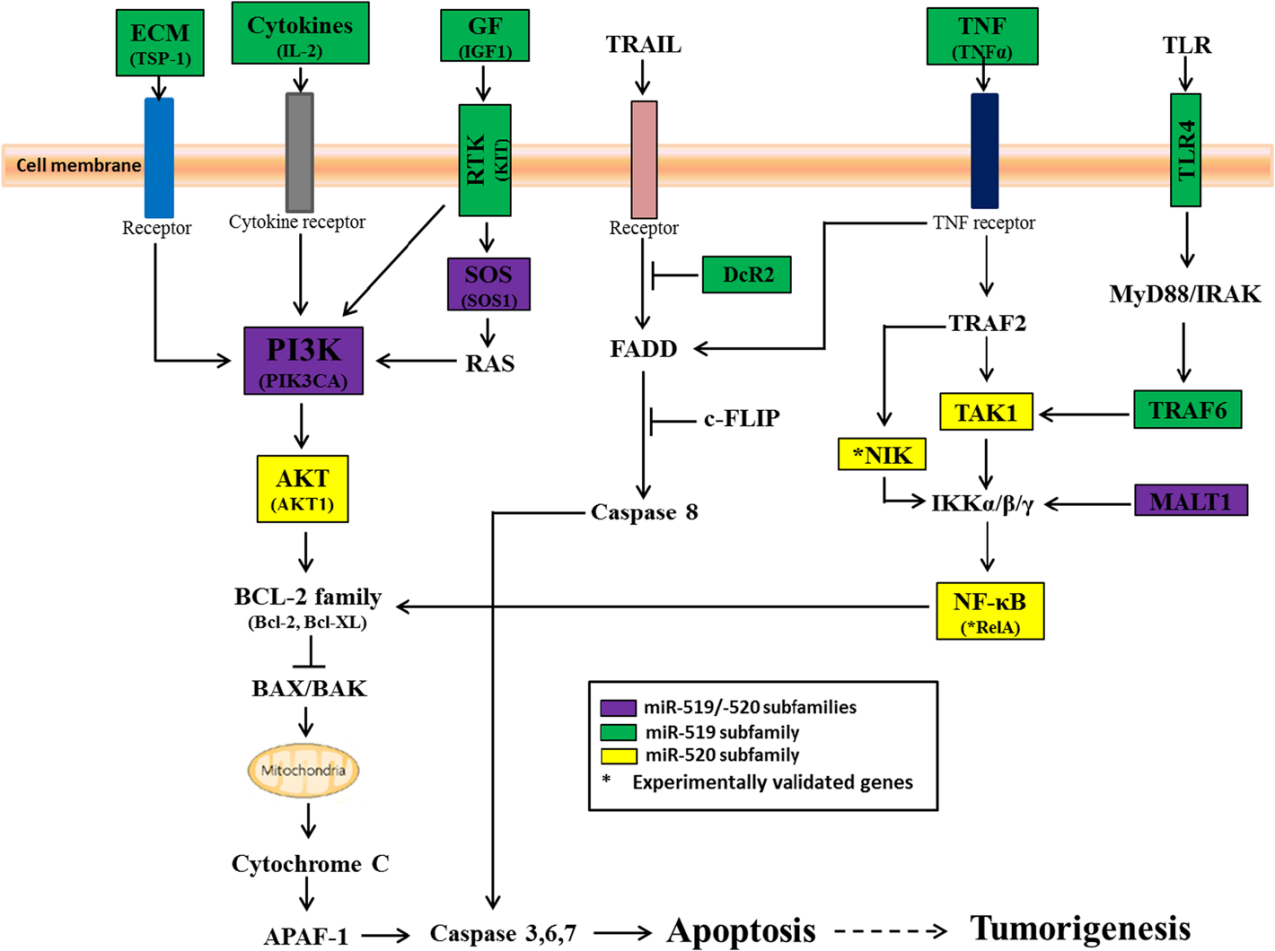
A proposed scheme that links the predicted group I C19MC-AAGUGC-miRNAs-targeted genes (in *color boxes*) to cell survival functions and apoptosis pathways. Genes targeted by either or both the miR-519 or -520 subfamilies are shown in different color boxes. See Discussion section for description of the proposed scheme

The regulatory pathways were further annotated by interrogation of the KEGG database, which yielded 24 pathways, which included 568 genes in total (Additional file 2: Table S2); 14 of the 24 KEGG pathways which may be related to pluripotency and cancer are shown in Fig. 3d. Ten of the 24 pathways, which included 260 (45.8%) genes, are different signaling pathways that are known to be involved in the growth and development processes [9, 10, 12, 21, 32–36]. Notably, 129 (22.7%) genes are associated with pathways regulating apoptosis including PI3K-AKT, MAPK, HIF-1 and TNF (Fig. 3d; see also Fig. 4 and Discussion below). The highest-enriched PI3K-Akt signaling pathway (56 genes) regulates cell survival by reducing apoptosis, stimulating cell growth and increasing proliferation [36]. Furthermore, many of the genes are related to pathways that regulate the cell cycle (22 genes) and apoptosis (13 genes) (Fig. 3d), which are important cellular events in the initiation and maintenance of stem cell pluripotency and tumorigenesis.

### Possible group I C19MC-AAGUGC-miRNAs targeting of the pro-apoptosis functions in the survival pathway

Suppression of apoptosis is an important feature of the initiate phase of the reprogramming process [37]. On the other hand, apoptosis dysregulation is associated with the different stages of tumorigenesis, including initiation, progression and metastasis [38]. A database search showed that the group I C19MC-AAGUGC-miRNAs target 179 apoptosis-associated genes (Additional file 3: Table S3). On the other hand, the KEGG pathway analysis above (Fig. 3d) has also revealed that the highest number of putative target genes of group I miRNAs are associated with PI3K-Akt, a survival pathway. Hence, we hypothesized that the group I miRNAs acted more specifically to inhibit apoptosis by targeting survival-related genes. Fifteen survival-related genes were predicted targets of the group I miRNAs (Table 4). Two out of the fifteen genes, *viz*. NIK and RelA, have been experimentally validated as direct targets miR-520e and miR-520c-3p [10, 12]. Importantly, the group I miRNAs may promote apoptosis either by indirectly activating pro-apoptotic proteins BAK/BAX through suppression of the cell survival-related genes [39], or by enhancing caspase-8 activation through targeting inhibitors of TRAIL-inducing apoptosis [40, 41]. Taken together, the group I C19MC-AAGUGC-miRNAs were predicted by bioinformatics analysis to regulate apoptosis, which is important in the initial phase of cellular reprogramming, and in particular the cell survival pathways, which are directly relevant to tumorigenesis processes.

**Table 4:** Predicted group I C19MC-AAGUGC-miRNA target genes associated with cell survival pathways

Gene Symbol	Gene name
AKT1	AKT serine/threonine kinase 1
IGF1	Insulin-like growth factor 1 (somatomedin C)
IL2	Interleukin 2
KIT	KIT proto-oncogene receptor tyrosine kinase
MALT1	Mucosa associated lymphoid tissue lymphoma translocation gene 1
NIK/MAP3K14^a^	Mitogen-activated protein kinase kinase kinase 14
PIK3CA	Phosphoinositide-3-kinase, catalytic, alpha polypeptide
RELA^a^	V-rel reticuloendotheliosis viral oncogene homolog A (avian)
SOS1	SOS Ras/Rac guanine nucleotide exchange factor 1
TAK1/MAP3K7	Nuclear receptor subfamily 2 group C member 2
TLR4	Toll-like receptor 4
TNF/TNFα	Tumor necrosis factor (TNF superfamily, member 2)
TNFRSF10D/DcR2	Tumor necrosis factor receptor superfamily, member 10d, decoy with truncated death domain
TRAF6	TNF receptor-associated factor 6
TSP-1/THBS1	Thrombospondin 1

^a^Experimentally validated target genes (Keklikoglou et al. [12]; Zhang et al. [10])

## Discussion

### Selective C19MC miRNA expression in MSC and in cancer cells suggests a complex transcriptional regulatory mechanism

In the present study and in the literature, data showed similar and disperse expression patterns of eight tested C19MC miRNAs in both mesenchymal stem and cancer cells (Fig. 1), in contrast to the previous model of *en bloc* expression in the choriocarcinoma JEG-3 cell line regulated by a master promoter [24]. Another studies have shown that the highly abundant Alu repetitive sequences embedded within the C19MC genomic region may function as independent RNA polymerase II promoters [42, 43]. Our study clearly showed selective C19MC miRNA activation in MSCs and HWP, and in cancer cells, suggesting that C19MC transcripts are more likely regulated by multiple promoters, which may in turn be active by condition-specific transcription factors. Furthermore in cancer cells, chromosomal rearrangements, amplification and modification of the promoter(s) or specific transcription factors could further regulate the selective C19MC miRNA expression. Previous reports have, indeed, shown that translocation of chromosomal band 19q13.4 selectively activated C19MC miRNAs in thyroid adenomas, and that C19MC genomic amplifications in an aggressive primitive neuroectodermal brain tumors were associated with specific and abundant expression of miR-517c and -520g [44]. Moreover, epigenetic alterations in the C19MC genomic region may also play important role in regulating C19MC expression, particularly in cancer and the dynamic stem cells. Promoter silencing of C19MC miRNAs by the DNA methylation inhibitor, 5-azacytidine, activated sixteen C19MC miRNAs [45]. Furthermore, placenta-derived mesenchymal stem cells were reported to escape epigenetic silencing of the paternal allele resulting in a number of the C19MC miRNAs being abundantly expressed [46]. Specific activation of the C19MC miR-512-5p by histone deacetylase inhibitors was also reported in human gastric cancer cells [43]. Transcription factors acting in *trans* are essential regulators of C19MC miRNA expression as shown by direct binding of p53 and the estrogen receptor α (ERα) to presumptive promoters of C19MC miR-519d and miR-515-5p, respectively, in chromatin immunoprecipitation assays [47, 48]. As a result of the specific DNA binding, miR-519d is up-regulated by p53, whereas ERα mediates both down- and up-regulated expression of miR-515-5p induced by estrogens and tamoxifen, respectively. Thus, transcription of specific C19MC miRNAs in MSC and cancer cells is likely highly complex, and may be dependent on the cellular and pathological state of the cells.

It was previously reported that C19MC is silenced in normal tissues [11, 25] due to hypermethylation of both the paternal and maternal alleles [26]. However, placenta is able to escape epigenetic silencing by maintaining paternal allele-expression [26, 45]. Moreover, the expression of miR-498, a member of C19MC, was reported in the fetal brain [49], echoing our report of Group C miRNAs being expressed in a fetal colon epithelium-derived cell line, CRL-1790 and placental Hs799. PI (Fig. 1c), consistent with C19MC expression in reproductive and developmental process-related tissues, relevant to the primate-specificity of the C19MC cluster.

In this study, we found that the 3p arms of the C19MC miRNA precursors were predominantly selected in ESCs and iPSCs (Table 2 and Fig. 2). Several studies have previously demonstrated that preferred arm selection is temporal- and spatial-dependent [50, 51]. Indeed, the 3p miRNA species have been shown to be more abundantly expressed in tumor tissues as opposed to preferred 5p selection in normal tissues [51]. Echoing these findings, the miR-302-like C19MC are also predominantly 3p-biased, possibly targeting genes which are biologically significant in regulating the stemness of stem cells and the tumor phenotype in cancers.

### Structural and function significance of the group I C19MC-AAGUGC-miRNAs

Our results also showed divergence in the positions of the AAGUGC seed sequence among C19MC miRNAs carrying the hexameric sequence. The canonical seed region situated at nucleotides 2-7 is a perfect seed match which markedly decreases the presence of false-positive bioinformatics predictions, thus improving prediction reliability [1]. Furthermore, the canonical seed region is crucial and sufficient to trigger target silencing [1]. The hexamer of half of the C19MC-AAGUGC-miRNAs reported here are located at nts 2-7, designated as group I C19MC-AAGUGC-miRNAs in this report (Fig. 2), suggesting high possibility that the predicted genes are the putative targets. Other non-canonical C19MC-AAGUGC-miRNAs are likely to have lower affinity and specificity and may be limited in mediating repression without the 3’-compensatory binding [52].

The group I miRNAs are composed of the miR-519 and -520 subfamilies. Despite their similar seed location at nts 2-7, Venn diagram analysis shows that these two subfamilies share only a small number of putative target genes (Fig. 3a). Common prediction algorithms that use identical powerful prediction characteristics, such as the mandatory stringent seed base-pairing produce different prediction results properly due to usage of various UTR databases as well as different internal criteria [53]. In this study, the putative target sets of the miR-519 and -520 subfamilies are overlapping gene sets predicted by two different prediction algorithms. Furthermore, one of the characteristics of target prediction, the sequence context surrounding the seed binding site of the target transcript [1], between the miR-519 and -520 subfamilies are also dissimilar (Fig. 2a). This may explain the different target gene sets of these two subfamilies. It has been reported that miR-96 and -182 that have identical seed region (UUGGCA, nucleotides 2-7) regulate different targets [54]. However, the miR-520 and miR-302/372 families share a significant number of target genes (Fig. 3a) suggesting common biological functions. Hence, it is highly likely that the group I miR-520 miRNAs may also contribute to reprogramming, as supported by the predicted involvement of miR-520 miRNAs in the reprogramming-related apoptosis and cell proliferation pathways (see Fig. 4 and Discussion below).

#### Regulation of C19MC miRNAs in tumorigenesis and stemness

Selective activation of C19MC miRNAs in MSCs and cancer cells reported here suggests functional involvement of the activated miRNAs in maintaining the stemness and promoting cancer development. Frequent aberrant C19MC miRNA expression in cancers has been reported [20, 44, 47]. Activation of the C19MC miR-519d was shown to target CDKN1A/p21, PTEN, AKT3 and TIMP2, and is closely associated with the pathogenesis of hepatocellular carcinoma by promoting cell proliferation and invasion, and in inhibiting apoptosis [47]. In breast cancer, high expression levels of plasma miR-520g is correlated with patients with lymph node metastasis and mammary gland invasion, and suppressed p53 expression [31].

On the other hands, C19MC miRNAs have also been shown to play important role in cellular stemness state. In normal embryonic development, many C19MC miRNAs have been shown to be expressed only in undifferentiated or germinal tissues, and C19MC expression inhibits differentiation of human embryonic stem cells [7, 26, 55]. The observation that the cellular reprogramming-able transcription factors OCT4 and NANOG regulate C19MC miRNA expression in human embryonic stem cells further supports close association of C19MC with induced pluripotency [56]. Moreover, the identification of sixteen miR-302-like C19MC miRNAs predicts functions in promoting “stemness” as the miR-302 and miR-372 families. Similarly, eight miR-302-like C19MC miRNAs were previously shown to promote cell proliferation and cell-cycle progression by targeting p21, an inhibitor of the G1/S transition, as for the miR-302 and -372 families [5, 6].

#### Possible involvement of group I C19MC-AAGUGC-miRNAs in regulating the apoptosis pathway common to stemness and cancer phenotype

Suppressed apoptosis is important to both the initial phase of acquiring pluripotency and in cancer progression [57, 58]. A combined expression profile and bioinformatics analysis reported in this work has, indeed, shown that the group I C19MC-AAGUGC-miRNAs**,** target genes related to the survival pathways (Table 4). Based on the predicted target genes, a scheme that correlates the group I C19MC-AAGUGC-miRNAs to stemness and cancer phenotype is proposed (Fig. 4). In general, group I miRNAs may enhance apoptosis through the PIK3/ATK, TNFs/NF-κB and TRAIL pathways, as predicted by KEGG pathway analysis (Fig. 4) [40, 58–60]. The PIK3 pathway is activated by a wide range of extracellular signals, including cytokines, e.g. IL-2 [61], growth factors, e.g. IGF1 [60] and components of the extracellular matrix (ECM) such as TSP-1 [62], all of which are the predicted targets of the group I miRNAs (Fig. 4). It is proposed here that the miRNAs target and inactivate the PIK3/AKT3 pathway by inhibition of the PIK3-related upstream genes TSP-1, IL-2, IGF1, KIT, SOS1 and PIK3CA, and the downstream AKT1 gene. The second important mechanism of cell survival is tumor necrosis factors (TNFs) activation of anti-apoptotic proteins via the nuclear factors of kappa B (NF-κB) signaling cascade (Fig. 4). Similar to the PIK3/ATK pathway, group I C19MC-AAGUGC- miRNAs may enhance apoptosis by the predicted targeting of the TNFα, TLR4, TRAF6, TAK1, NIK, MALT1 and RelA genes. Thirdly, group I miRNAs are also predicted to silence genes, such as DcR2, that are inhibitory to the TRAIL-induced apoptosis pathway, resulting in pro-apoptosis [40]. The group I miRNAs-modulated pathways subsequently suppress the activation of downstream effector caspase-3, -6, and -7, thus inhibiting apoptosis and promoting proliferation [63].

## Conclusions

In the present study, the data show selective expression of C19MC miRNAs in cancer and stem cells, offering insights into possible involvement of selective C19MC miRNAs in regulation of “stemness” and tumorigenesis, possibly via the cell survival pathways. More specifically, a subgroup of sixteen C19MC miRNAs has been identified that shares the same AAGUGC seed sequence as the reprogramming miR-302/372 family, predicting contribution of the C19MC-AAGUGC-miRNAs to the reprograming process. Further elucidation of the biological functions of C19MC miRNAs, particular the miRNA-302-like subclass, may lead to potential applications in more efficient cellular reprogramming and in cancer therapy.

## Additional files

Additional file 1: Table S1. List of putative target genes. (XLSX 60 kb)
Additional file 2: Table S2. KEGG pathways of the Group I C19MC miRNAs. (DOCX 14 kb)
Additional file 3: Table S3. Predicted target genes of group I C19MC miRNAs related to apoptosis. (DOCX 22 kb)

## Abbreviations

C19MC: The human chromosomal 19 miRNA cluster
ERα: Estrogen receptor α
ESC: Embryonic stem cells
GO: Gene ontology
HCT15 and SKCO1: Colorectal cancer
HepG2: Liver cancer
HWP: Human white pre-adipocyte
iPSC: Induced pluripotent stem cells
JEG-3: Human placental choriocarcinoma cell line
KEGG: Kyoto Encyclopedia of Genes and Genomes
MCF-7: Breast cancer
miRNAs: microRNAs
MSCs: Mesenchymal stem cells
