# Supplementary material for: Selective activation of miRNAs of the primate-specific chromosome 19 miRNA cluster (C19MC) in cancer and stem cells and possible contribution to regulation of apoptosis
Source: J Biomed Sci. 2017 Mar 7;24:20. doi: 10.1186/s12929-017-0326-z (PMC5341377; doi:10.1186/s12929-017-0326-z)
Supplement: Additional file 2: Table S2. — KEGG pathways of the Group I C19MC miRNAs. (DOCX 14 kb) [file 12929_2017_326_MOESM2_ESM.docx]

**Suppl Table S2** KEGG pathways of the Group I C19MC-AAGUGC-miRNAs

| No | KEGG designation | Pathway | No. target genes |
| --- | --- | --- | --- |
| **1** | **hsa04151** | **PI3K-Akt signaling pathway** | **56** |
| 2 | hsa05200 | Pathways in cancer | 54 |
| 3 | hsa04144 | Endocytosis | 39 |
| **4** | **hsa04010** | **MAPK signaling pathway** | **38** |
| **5** | **hsa04014** | **Ras signaling pathway** | **37** |
| 6 | hsa05205 | Proteoglycans in cancer | 30 |
| 7 | hsa05162 | Measles | 24 |
|  | **hsa04068** | **FoxO signaling pathway** | **24** |
| 9 | hsa04110 | Cell cycle | 22 |
|  | hsa04360 | Axon guidance | 22 |
|  | hsa04380 | Osteoclast differentiation | 22 |
| **12** | **hsa04350** | **TGF-beta signaling pathway** | **19** |
|  | **hsa04152** | **AMPK signaling pathway** | **19** |
| **14** | **hsa04066** | **HIF-1 signaling pathway** | **18** |
|  | **hsa04660** | **T cell receptor signaling pathway** | **18** |
|  | hsa04931 | Insulin resistance | 18 |
| **17** | **hsa04668** | **TNF signaling pathway** | **17** |
| 18 | hsa00564 | Glycerophospholipid metabolism | 16 |
| **19** | **hsa04662** | **B cell receptor signaling pathway** | **14** |
|  | hsa05133 | Pertussis | 14 |
|  | hsa04612 | Antigen processing and presentation | 14 |
| 22 | hsa04210 | Apoptosis | 13 |
| 23 | hsa05134 | Legionellosis | 11 |
| 24 | hsa03030 | DNA replication | 9 |
| **Total** | | | **568** |

The ten signaling pathways are shown in bold letters.
