# Supplementary material for: Selective activation of miRNAs of the primate-specific chromosome 19 miRNA cluster (C19MC) in cancer and stem cells and possible contribution to regulation of apoptosis
Source: J Biomed Sci. 2017 Mar 7;24:20. doi: 10.1186/s12929-017-0326-z (PMC5341377; doi:10.1186/s12929-017-0326-z)
Supplement: Additional file 3: Table S3. — Predicted target genes of group I C19MC miRNAs related to apoptosis. (DOCX 22 kb) [file 12929_2017_326_MOESM3_ESM.docx]

**Supp. Table S3** Predicted target genes of group I C19MC-AAGUGC- miRNAs related to apoptosis

| **Gene ID** | **Gene name** |
| --- | --- |
| ACTN2 | Actinin, alpha 2 |
| AHI1 | Abelson helper integration site 1 |
| AIFM1 | Apoptosis-inducing factor, mitochondrion-associated, 1 |
| **AKT1** | AKT serine/threonine kinase 1 |
| AKTIP | AKT interacting protein |
| ALX4 | ALX homeobox 4 |
| AMIGO2 | Adhesion molecule with Ig-like domain 2 |
| APH1A | Anterior pharynx defective 1 homolog A (C. elegans) |
| API5 | API5-like 1; apoptosis inhibitor 5 |
| AQP1 | Aquaporin 1 (Colton blood group) |
| AREL1 | Apoptosis resistant E3 ubiquitin protein ligase 1 |
| ARHGEF11 | Rho guanine nucleotide exchange factor (GEF) 11 |
| ARHGEF17 | Rho guanine nucleotide exchange factor (GEF) 17 |
| ARHGEF18 | Rho/Rac guanine nucleotide exchange factor (GEF) 18 |
| ARL6IP1 | ADP-ribosylation factor-like 6 interacting protein 1 |
| ATM | ATM serine/threonine kinase |
| BCAP29 | B-cell receptor-associated protein 29 |
| BCL10 | B-cell CLL/lymphoma 10 |
| BCL2L15 | BCL2-like 15 |
| BCL6 | B-cell CLL/lymphoma 6 |
| BCLAF1 | Similar to Bcl-2-associated transcription Factor 1 (Btf); BCL2-associated transcription factor 1 |
| BECN1 | Beclin 1, autophagy related |
| BIRC5 | baculoviral IAP repeat containing 5 |
| BIRC6 | Baculoviral IAP repeat-containing 6 |
| BMP6 | Bone morphogenetic protein 6 |
| BMP8B | Bone morphogenetic protein 8b |
| BNIP1 | BCL2/adenovirus E1B 19kDa interacting protein 1 |
| BNIP3L | BCL2/adenovirus E1B 19kDa interacting protein 3-like |
| C3ORF38 | Chromosome 3 open reading frame 38 |
| CAAP1 | Caspase activity and apoptosis inhibitor 1 |
| CARD18 | Caspase recruitment domain family, member 18 |
| CASP7 | Caspase 7, apoptosis-related cysteine peptidase |
| CASP8 | CASP8 and FADD-like apoptosis regulator |
| CCND2 | Cyclin D2 |
| CD44 | CD44 molecule (Indian blood group) |
| CDCA7 | Cell division cycle associated 7 |
| CDKN1A | Cyclin-dependent kinase inhibitor 1A (p21, Cip1) |
| CFLAR | CASP8 and FADD like apoptosis regulator |
| CHMP3 | Charged multivesicular body protein 3 |
| CHST11 | Carbohydrate (chondroitin 4) sulfotransferase 11 |
| CIDEA | Cell death-inducing DFFA-like effector a |
| CSRNP3 | Cysteine-serine-rich nuclear protein 3 |
| CXCR4 | Chemokine (C-X-C motif) receptor 4 |
| DAB2 | DAB2, clathrin adaptor protein |
| DAD1 | Defender against cell death 1 |
| DAPK2 | Death-associated protein kinase 2 |
| **DcR2/TNFRSF10D** | Tumor necrosis factor receptor superfamily, member 10d, decoy with truncated death domain |
| DDIAS | DNA damage-induced apoptosis suppressor |
| DEDD2 | Death effector domain containing 2 |
| DLG5 | Discs large MAGUK scaffold protein 5 |
| DNAJC3 | DnaJ (Hsp40) homolog, subfamily C, member 3 |
| DNM1L | Dynamin 1-like |
| DRAM1 | DNA-damage regulated autophagy modulator 1 |
| DRAM2 | DNA-damage regulated autophagy modulator 2 |
| ECT2 | Epithelial cell transforming 2 |
| EDNRB | Endothelin receptor type B |
| EFNA5 | Ephrin-A5 |
| EGLN3 | Egl-9 family hypoxia-inducible factor 3 |
| ERBB4 | Erb-b2 receptor tyrosine kinase 4 |
| ESR1 | Estrogen receptor 1 |
| FASLG | Fas ligand (TNF superfamily, member 6) |
| FBXO10 | F-box protein 10 |
| FCMR | Fc fragment of IgM receptor |
| FGD4 | FYVE, RhoGEF and PH domain containing 4 |
| FIGNL1 | Fidgetin-like 1 |
| FLT4 | Fms-related tyrosine kinase 4 |
| FOXL2 | Forkhead box L2 |
| GADD45B | Growth arrest and DNA-damage-inducible, beta |
| GDF11 | Growth differentiation factor 11 |
| GHITM | Growth hormone inducible transmembrane protein |
| GJA1 | Gap junction protein, alpha 1, 43kDa |
| GLO1 | Glyoxalase I |
| GPLD1 | Glycosylphosphatidylinositol specific phospholipase D1 |
| HIF3A | Hypoxia inducible factor 3, alpha subunit |
| HIGD1A | HIG1 hypoxia inducible domain family, member 1A |
| HIP1 | Huntingtin interacting protein 1 related |
| HIP1R | Huntingtin interacting protein 1 related |
| HIPK3 | Homeodomain interacting protein kinase 3 |
| **IGF1** | Insulin-like growth factor 1 (somatomedin C) |
| **IL2** | Interleukin 2 |
| INHBC | Inhibin, beta C |
| IRF1 | Interferon regulatory factor 1 |
| IRF5 | Interferon regulatory factor 5 |
| ITGB2 | Integrin, beta 2 |
| KIF14 | Kinesin family member 14 |
| **KIT** | KIT proto-oncogene receptor tyrosine kinase |
| KLHL20 | Kelch-like family member 20 |
| KLLN | Killin, p53-regulated DNA replication inhibitor |
| LEF1 | Lymphoid enhancer-binding factor 1 |
| LEFTY1 | Left-right determination factor 1 |
| LEFTY2 | Left-right determination factor 2 |
| LHX3 | LIM homeobox 3 |
| MAGEH1 | Melanoma antigen family H1 |
| **MALT1** | Mucosa associated lymphoid tissue lymphoma translocation gene 1 |
| MAP3K5 | Mitogen-activated protein kinase kinase kinase 5 |
| MAP3K8 | Mitogen-activated protein kinase kinase kinase 8 |
| MAP3K9 | Mitogen-activated protein kinase kinase kinase 9 |
| MCL1 | Myeloid cell leukemia sequence 1 (BCL2-related) |
| MCM2 | Minichromosome maintenance complex component 2 |
| MDM4 | MDM4, p53 regulator |
| MEF2C | Myocyte enhancer factor 2C |
| MFN2 | Mitofusin 2 |
| MTCH2 | Mitochondrial carrier 2 |
| **NIK/MAP3K14** | Mitogen-activated protein kinase kinase kinase 14 |
| NLRP3 | NLR family, pyrin domain containing 3 |
| NOD1 | Nucleotide-binding oligomerization domain containing 1 |
| NOX4 | NADPH oxidase 4 |
| NPM1 | Nucleophosmin |
| NR2E1 | Nuclear receptor subfamily 2, group E, member 1 |
| OGT | O-linked N-acetylglucosamine (GlcNAc) transferase |
| PAFAH2 | Platelet-activating factor acetylhydrolase 2 |
| PAK6 | P21 protein (Cdc42/Rac)-activated kinase 6 |
| PAX8 | Paired box 8 |
| PDCD2 | Programmed cell death 2 |
| PDCD4 | Programmed cell death 4 |
| **PIK3CA** | Phosphoinositide-3-kinase, catalytic, alpha polypeptide |
| PKN2 | Protein kinase N2 |
| PLAC8 | Placenta-specific 8 |
| PLAUR | Plasminogen activator, urokinase receptor |
| PRKAA1 | Protein kinase, AMP-activated, alpha 1 catalytic subunit |
| PRNP | Prion protein |
| PTK2 | Protein tyrosine kinase 2 |
| PUF60 | Poly-U binding splicing factor 60KDa |
| RBM25 | RNA binding motif protein 25 |
| **RELA** | V-rel reticuloendotheliosis viral oncogene homolog A (avian) |
| RNF216 | Ring finger protein 216 |
| RPS6KA1 | Ribosomal protein S6 kinase, 90kDa, polypeptide 1 |
| RPS6KA3 | Ribosomal protein S6 kinase, 90kDa, polypeptide 3 |
| SAP18 | Sin3A-associated protein, 18kDa |
| SERPINB9 | Serpin peptidase inhibitor, clade B (ovalbumin), member 9 |
| SFRP4 | Secreted frizzled-related protein 4 |
| SGMS1 | Sphingomyelin synthase 1 |
| SHC4 | SHC (Src homology 2 domain containing) family, member 4 |
| SLC40A1 | Solute carrier family 40 (iron-regulated transporter), member 1 |
| SLTM | SAFB-like, transcription modulator |
| SMNDC1 | Survival motor neuron domain containing 1 |
| SNCA | Synuclein, alpha |
| SOD1 | Superoxide dismutase 1, soluble |
| SOD2 | Superoxide dismutase 2, mitochondrial |
| **SOS1** | SOS Ras/Rac guanine nucleotide exchange factor 1 |
| SOX4 | SRY (sex determining region Y)-box 4 |
| SQSTM1 | Sequestosome 1 |
| STAT3 | signal transducer and activator of transcription 3 |
| STK17B | Serine/threonine kinase 17b |
| STK4 | Serine/threonine kinase 4 |
| **TAK1/MAP3K7** | Mitogen-activated protein kinase kinase kinase 7 |
| TAOK2 | TAO kinase 2 |
| TAX1BP1 | Tax1 (human T-cell leukemia virus type I) binding protein 1 |
| TFAP4 | Transcription factor AP-4 (activating enhancer binding protein 4) |
| TGFBR2 | Transforming growth factor, beta receptor II |
| TGM2 | Transglutaminase 2 |
| TIAM1 | T-cell lymphoma invasion and metastasis 1 |
| TIGAR | TP53 induced glycolysis regulatory phosphatase |
| **TLR4** | Toll-like receptor 4 |
| TMBIM6 | Transmembrane BAX inhibitor motif containing 6 |
| **TNF/TNFα** | Tumor necrosis factor (TNF superfamily, member 2) |
| TNFAIP1 | Tumor necrosis factor, alpha-induced protein 1 (endothelial) |
| TNFRSF10B | Tumor necrosis factor receptor superfamily, member 10b |
| TNFRSF19 | Tumor necrosis factor receptor superfamily, member 19 |
| TNFRSF21 | Tumor necrosis factor receptor superfamily, member 21 |
| TNFRSF8 | Tumor necrosis factor receptor superfamily, member 8 |
| TOPORS | Topoisomerase I binding, arginine/serine-rich, E3 ubiquitin protein ligase |
| TOX3 | TOX high mobility group box family member 3 |
| TP73 | Tumor protein p73 |
| TRAF4 | TNF receptor-associated factor 4 |
| **TRAF6** | TNF receptor-associated factor 6 |
| TRIM24 | Tripartite motif containing 24 |
| **TSP-1/THBS1** | Thrombospondin 1 |
| TXNIP | Thioredoxin interacting protein |
| UBC | Ubiquitin C |
| UNC5C | Unc-5 netrin receptor C |
| UNC5D | Unc-5 netrin receptor D |
| USP47 | Ubiquitin specific peptidase 47 |
| VAV3 | Vav 3 guanine nucleotide exchange factor |
| VCP | Valosin containing protein |
| WDR92 | WD repeat domain 92 |
| ZNF385A | Zinc finger protein 385A |
| ZNF443 | Zinc finger protein 443 |
| ZNF830 | Zinc finger protein 830 |

*Bold genes: Genes related to survival pathway
